# OR3-001 – RIP2 kinase is activated in Blau Syndrome and IBD

**DOI:** 10.1186/1546-0096-11-S1-A3

**Published:** 2013-11-08

**Authors:** KP Foley, B Desai, A Vossenkämper, MA Reilly, P Biancheri, L Wang, DB Lipshutz, J Connor, M Miller, PA Haile, LN Casillas, BJ Votta, PJ Gough, TT MacDonald, CH Wouters, CD Rosé, J Bertin

**Affiliations:** 1 Pattern Recognition Receptor Discovery Performance Unit, GlaxoSmithKline, Collegeville, United States; 2 Barts and The London School of Medicine and Dentistry, London, United Kingdom; 3 University of Leuven, Leuven, Belgium; 4 Alfred I. duPont Hospital for Children, Wilmington, United States

## Introduction

Blau Syndrome (Blau) is a granulomatous auto-inflammatory disease caused by mutations in NOD2 that have been proposed to result in phosphorylation of RIP2 kinase and the production of pro-inflammatory cytokines. Such monogenic diseases can bring to light pathways that are also likely to be involved in more genetically complex diseases. For example, increased RIP2 phosphorylation has been observed in inflammatory bowel disease (IBD), although the role of RIP2 in IBD has not been determined. We are developing a first-in-class, highly potent and selective inhibitor of RIP2 kinase, which may provide therapeutic benefit in both Blau and IBD.

## Objectives

To explore the role of RIP2 in disease, we examined its phosphorylation state in peripheral blood mononuclear cells (PBMCs) and synovial fluid (SF) cells isolated from a cohort of Blau patients. We also assessed the ability of a RIP2 inhibitor to block inflammatory cytokine production in *ex vivo*-cultured mucosal biopsies from IBD patients.

## Methods

Phospho-Serine176-RIP2 (pRIP2) levels were measured in PBMCs from 5 Blau patients and 4 normal healthy volunteers (NHVs) and SF cells from the inflamed knee joints of 1 Blau patient by immunoblotting with a novel monoclonal antibody. Inflamed biopsies from 28 IBD patients were cultured *ex vivo* in serum-free media for 18 hrs in the presence or absence of RIP2 inhibitor GSK’214, and culture supernatants were assayed for TNF-α, IL-1β and IL-6 by ELISA.

## Results

pRIP2 levels were found to be elevated by an average of 8-fold in PBMCs isolated from Blau patients relative to NHVs. SF cells from a Blau patient also expressed 240-fold higher levels of pRIP2 than NHV PBMCs. Studies are underway to assess the effect of RIP2 inhibition on pRIP2 in cultured PBMCs from Blau patients.

Treatment with RIP2 inhibitor GSK’214 resulted in dose-dependent inhibition of pRIP2 in *ex vivo*-cultured IBD biopsies. GSK’214 also inhibited spontaneous production of TNF-α, IL-1β and IL-6 in these cultures, with efficacy equivalent to that of prednisolone or dexamethasone. Similar results were observed with biopsies from both Crohn’s disease and ulcerative colitis patients.

## Conclusion

Studies in transfected cells have suggested that Blau NOD2 mutations act in a gain-of-function manner, however, direct evidence for the activation of RIP2 in patients has been lacking. We have for the first time shown that increased phosphorylation of RIP2 on Serine176, a well-established phosphorylation site associated with kinase activation, occurs in primary Blau patient cells, confirming the dominant gain-of-function nature of these mutations. Prompted by reports of increased pRIP2 in mucosal tissues from IBD patients, we have also shown that inhibition of RIP2 activation suppresses the spontaneous production of inflammatory cytokines by *ex vivo*-cultured IBD biopsies. Our results suggest that both Blau and IBD patients may benefit from therapeutic inhibition of RIP2.

## Disclosure of interest

K. Foley Employee of: GlaxoSmithKline, B. Desai Employee of: GlaxoSmithKline, A. Vossenkämper Grant / Research Support from: GlaxoSmithKline, M. Reilly Employee of: GlaxoSmithKline, P. Biancheri: None Declared, L. Wang Employee of: GlaxoSmithKline, D. Lipshutz Employee of: GlaxoSmithKline, J. Connor Employee of: GlaxoSmithKline, M. Miller Employee of: GlaxoSmithKline, P. Haile Employee of: GlaxoSmithKline, L. Casillas Employee of: GlaxoSmithKline, B. Votta Employee of: GlaxoSmithKline, P. Gough Employee of: GlaxoSmithKline, T. MacDonald Grant / Research Support from: GlaxoSmithKline, C. Wouters Grant / Research Support from: GlaxoSmithKline, C. Rosé Grant / Research Support from: GlaxoSmithKline, J. Bertin Employee of: GlaxoSmithKline.

